# Different Life Cycle Stages of *Plasmodium falciparum* Induce Contrasting Responses in Dendritic Cells

**DOI:** 10.3389/fimmu.2019.00032

**Published:** 2019-01-31

**Authors:** Xi Zen Yap, Rachel J. Lundie, Gaoqian Feng, Joanne Pooley, James G. Beeson, Meredith O'Keeffe

**Affiliations:** ^1^Burnet Institute, Melbourne, VIC, Australia; ^2^Department of Medicine, The University of Melbourne, Parkville, VIC, Australia; ^3^Department of Biochemistry and Molecular Biology and Biomedicine Discovery Institute, Monash University, Clayton, VIC, Australia; ^4^Department of Microbiology, Monash University, Clayton, VIC, Australia; ^5^Central Clinical School, Monash University, Melbourne, VIC, Australia

**Keywords:** dendritic cell (DC), malaria, malaria vaccines, parasite-host interactions, innate immunity

## Abstract

Dendritic cells are key linkers of innate and adaptive immunity. Efficient dendritic cell activation is central to the acquisition of immunity and the efficacy of vaccines. Understanding how dendritic cells are affected by *Plasmodium falciparum* blood-stage parasites will help to understand how immunity is acquired and maintained, and how vaccine responses may be impacted by malaria infection or exposure. This study investigates the response of dendritic cells to two different life stages of the malaria parasite, parasitized red blood cells and merozoites, using a murine model. We demonstrate that the dendritic cell responses to merozoites are robust whereas dendritic cell activation, particularly CD40 and pro-inflammatory cytokine expression, is compromised in the presence of freshly isolated parasitized red blood cells. The mechanism of dendritic cell suppression by parasitized red blood cells is host red cell membrane-independent. Furthermore, we show that cryopreserved parasitized red blood cells have a substantially reduced capacity for dendritic cell activation.

## Introduction

Dendritic cells (DCs) are key linkers of innate and adaptive immunity and their activation is essential to vaccination. Ablating DCs in murine models leads to vaccination failure and subsequent loss of antigen-specific T cells (1). Immunization of individuals living in malaria-infested regions has been notoriously difficult, with even the most promising vaccines failing to provide high levels of efficacy. A failure to activate DCs in people infected with *Plasmodium falciparum* blood-stage parasites may provide some explanation to the low efficacy of malaria vaccines [reviewed in (2)]. It may also explain the slow acquisition of natural anti-malarial immunity in individuals residing in malaria-endemic settings.

Activation of DCs is required for their subsequent efficient activation of T cells. Activated DCs increase surface expression of the co-stimulatory markers MHCII, CD40, CD80, and CD86, which are all involved in T cell co-stimulation. In particular, the interaction between CD40 on DC and its ligand CD40L on T cells is integral to enhance both T cell and DC responses for effective adaptive immune responses (3).

There remains an unresolved question of whether exposure to *P. falciparum* inhibits DC activation, leading to DC “suppression.” Literature from *ex vivo* studies has suggested that DCs from infected individuals are inhibited in core DC functions, namely: expression of co-stimulatory markers, cytokine secretion, antigen uptake, and the ability to induce primary and secondary T cell responses (4–7). However, there is a lack of consensus among *in vitro* studies as to what effect *P. falciparum* has directly upon DCs. To date the majority of literature has examined DC responses to parasitized erythrocytes (pRBCs), the intracellular stage of the asexual parasite. In this life stage *P. falciparum* is concealed from host immunity by the host erythrocyte membrane, though parasite proteins such as the adhesion ligand *PfEMP1* are exported to the erythrocyte surface. Very little research into cellular responses to the infectious extracellular stage, known as the merozoite, has been carried out. Furthermore, it is important to note that the majority of *in vitro* studies have been carried out in monocyte-derived (mo) DCs (8), which are phenotypically and functionally different from *bona fide* DCs (8, 9).

The murine Flt3 ligand-induced DCs (FL-DCs) closely recapitulate the DCs of the blood, similar to spleen DC, but slightly less mature conventional (cDC) and plasmacytoid (pDC) DC subsets (10–13). Therefore, the FL-DC model was used here for its ease and its capacity to generate large volumes of *bona fide* DCs, enabling us to test a broad variety of parameters.

The FL-DC cDCs can be divided into two subsets by expression of CD24 and CD11b. The FL-DC CD24^+^ cDCs (CD24^+^CD11b^−^) are immature cDC1. The cDC1 subset is specialized in cross-presentation and has a superior ability to prime naive cytotoxic CD8^+^ T cells, and polarize T cells toward the T_H_1 pathway via IL-12 production. Meanwhile, the FL-DC CD11b^+^ subset (CD24^−^CD11b^+^) is equivalent to immature cDC2 (11). These FL-DC cDC1 and cDC2 populations share many pattern recognition receptors with their human blood DC counterparts and the core surface phenotypes and functions of cross-presentation (cDC1) and bacterial recognition and CD4^+^ T cell stimulation (cDC2) are conserved [reviewed in Macri et al. (14)].

In contrast, pDCs are overall poor stimulators of T cell responses and do not upregulate co-stimulatory markers. Their defining characteristic is the secretion of Type I and III interferons (IFNs) in response to viral infection and/or the ligation of endosomal TLRs (15–17). In humans, pDCs are also the only subset to express TLR9, which is a known receptor for malarial DNA (18, 19).

Previous literature has described pDC production of IFN-α in response to *P. falciparum* merozoites *in vitro* (19) and during natural infection in humans (20), where pDCs appear suppressed in the presence of malaria parasites (21). Further studies hinted at pDC as a source of replicating malaria parasites (22), but this subset has, overall, been poorly studied in malaria.

This study aimed to identify how DC responses to the pRBC and the merozoite differ, with findings indicating that merozoites induce DC activation whereas pRBCs induce low co-stimulatory marker expression but high production of cytokines, with greater suppression at increasing doses of pRBCs. We reveal that the mechanism of suppression of DC activation occurs independently of the host RBC membrane, is not seen with pRBCs that have undergone a freeze-thaw cycle, and cannot be rescued by merozoites nor an exogenous TLR9 stimulus.

## Methods

### Culture, Purification, and Freeze-Thawing of *P. falciparum* Blood-Stage Parasites

*Plasmodium falciparum* 3D7 parasites were cultured as per standard protocols. Briefly, parasites were maintained in RPMI1640 (Gibco) enriched with 5% Albumax (Gibco) and 5% AB^+^ human serum obtained from the Red Cross, Melbourne, Australia. Parasites were cultured at 3–5% haematocrit using type-O negative blood (Red Cross) and incubated in medical gas (1% O_2_ and 5% CO_2_ in nitrogen gas) at 37°C as per standard protocols. Parasite growth was assessed daily by thin smear (23) stained with Giemsa's azur eosin methylene blue solution (Merck Millipore). Parasites were sorbitol synchronized (24) and selected for knob expression by gelatin flotation (25). All parasites were initially treated with *Mycoplasma* removal agent (MP Biomedicals) for 2 weeks as per the manufacturer's instructions. Subsequently, parasites were tested at 1-monthly intervals for *Mycoplasma* contamination using the MycoAlert test kit (Lonza).

### Trophozoite Preparation

Trophozoites at 24–28 h post-invasion were isolated by magnetic selection with the VarioMACS (Miltenyi Biotech) system. Purity was assessed by counting 200 cells via Giemsa thin smear. Preparations varied from 70 to 99% parasite purity and preparations with purity of < 90% were re-purified by repeating magnet selection. Purified preparations were counted using a haemocytometer and resuspended to the appropriate cell concentration in complete medium (CM): RPMI1640 containing GlutaMAX (Gibco) supplemented with 10% heat-inactivated FCS, 0.1 mM 2-beta-mercaptoethanol, 100 U/mL penicillin and 100 μg/mL streptomycin. Aliquots of purified trophozoites with >95% purity were also frozen in glycerolyte 57 (Baxter Healthcare Corporation) and stored at −80°C to compare efficacy of DC stimulation with same-day isolated cultures.

### Merozoite Preparation

Merozoites were purified as per Boyle et al. (26). Briefly, late-stage (30–34 h) trophozoites were purified and returned to culture without uninfected RBCs (uRBCs). When trophozoites had matured into schizonts (40 h), 10 μM of protease inhibitor E64 (Sigma) was added to culture medium to inhibit merozoite egress. Upon maturation into late-stage schizonts (46–48 h), pRBCs were resuspended in 20 mL of RPMI-HEPES and schizont rupture was induced by pushing through a 1.2 μM syringe filter (Pall), resulting in purified merozoites. Merozoites were stained with ethidium bromide (EtBr) and counted by flow cytometry. For storage, late-stage segmented schizonts derived by this method were frozen in PBS at a concentration of 150 × 10^7^/mL after arrest with E64 but prior to filter lysis and stored at −80°C until use in assays.

### Saponin Lysis to Remove Host RBC Membranes

Magnet-purified pRBCs or empty “mock” tubes were co-incubated with 0.09% saponin in 1X PBS for 30 s at RT, after which they were washed three times in RPMI containing 2% FCS to remove residual saponin. Saponin lysis of host RBC membrane was confirmed by microscopy. Both saponin-treated and intact parasites were then resuspended in CM and co-incubated with FL-DCs in the presence or absence of 0.5 μM CpG2216. CM or CM and CpG2216 were added to “mock” tubes to control for any potential carry-over of the detergent and added to FL-DCs as separate controls.

### FL-DC Culture

Bone marrow cells were stimulated with Flt3-L to induce large quantities of DCs similar in phenotype and function to those found in murine spleen (11, 12, 27) (Supplementary Figure 1). Briefly, FL-DCs were obtained as follows: leg bones of C57Bl/6 mice (AMREP Animal Services Precinct Animal Centre, Melbourne, Australia) were dissected out and harvested into RPMI containing 2% v/v FCS. Bones were cut open and bone marrow was flushed with a 22 g needle using 2 mL of RPMI-FCS per bone. After centrifugation at 600 × *g* for 7 min, cells were co-incubated with 1 mL of red cell lysis buffer (Sigma) with constant mixing for 30 s. Cells were washed twice in RPMI1640 containing 2% FCS before resuspending in complete medium and filtering through a 70 μM sieve (Falcon). Cells were resuspended at 1.5 × 10^6^ cells/mL in complete medium containing 100 ng/mL of Flt3-L-Ig (BioXCell) and cultured in vented cap tissue culture flasks (Corning) in a 37°C incubator in 5% CO_2_ for 8 days.

### Determining the Effect of the Parasite-DC Ratio Upon the DC Response to Purified Blood-Stage *P. falciparum*

After 8 days culture FL-DC were counted and washed in CM. Merozoites and pRBCs were titrated against FL-DCs in round-bottom 96-well-tissue culture plates (Falcon) at a final cell concentration of 0.5 × 10^6^ FL-DC/mL. Additionally, FL-DCs were also cultured with 0.5 μM CpG2216 alone or in addition to parasites, or with media alone or uRBCs as negative controls. All incubations were carried out for 18–20 h at 37°C in 5% CO_2_.

### Flow Cytometric Analysis

For analysis of surface phenotype, cells were stained in 1X PBS supplemented with 2% v/v FCS and 10% v/v 0.1M EDTA (MACS buffer) with monoclonal antibodies for MHCII, CD11b, CD11c, CD24, CD45R, and CD199. For staining, antibody was diluted in MACS buffer and antibody cocktail was added at 10μL/10^6^ cells, with a minimum of 30 μL of cocktail per well or tube, and incubated in the dark at 4°C for 20 min. Cells were washed to remove unbound antibody and analyzed on a BD Fortessa or BD LSRII flow cytometer. DCs were identified as medium-sized cells on forward and side scatter and doublets and PI-positive dead cells excluded. Activation of viable DCs was measured by expression of costimulatory markers MHCII, CD40, CD69, CD80, and CD86 on each DC subset using antibodies outlined in Supplementary Table 1.

### Cytokine Analysis

Frozen supernatants derived from parasite-FL-DC co-culture experiments were tested for the presence of IFN-γ, IL-1β, IL-2; IL-4, IL-5, IL-9, IL-12p70, IL-13, IL-17a, IL-18, IL-22, IL-23, IL-27, GM-CSF, IP-10, eotaxin, and MIP-1α by ProcartaPlex (eBioscience) multiplex as per manufacturer's instructions. Presence of MIP-2, IL-6, IL-10, GRO-α, MCP-1, MCP-3, MIP-1β, RANTES, and TNF-α were assayed for by both FlowCytomix (eBioScience) and ProcartaPlex. Expression of IFN-α and IFN-β was measured by a separate multiplex (ProcartaPlex).

## Results

### The Ratio of pRBCs-to-DCs Affects Expression of DC Maturation Markers and This Is Not Rescued in the Presence of TLR9 Ligand

People in endemic malaria regions can have a wide range of parasitemia in peripheral blood. Those with asymptomatic malaria typically have lower levels of parasitemia, ~5,000 *P. falciparum* parasites per mL of peripheral blood on average (28), depending on the setting. This equates to a ratio of ~0.25 parasites to 1 DC in blood. However, significant numbers of asymptomatic people and those with symptomatic malaria have infections over 10^6^ parasites per mL, equating to ~50:1 ratio of parasites to blood DC (29, 30).

**Figure 1:**
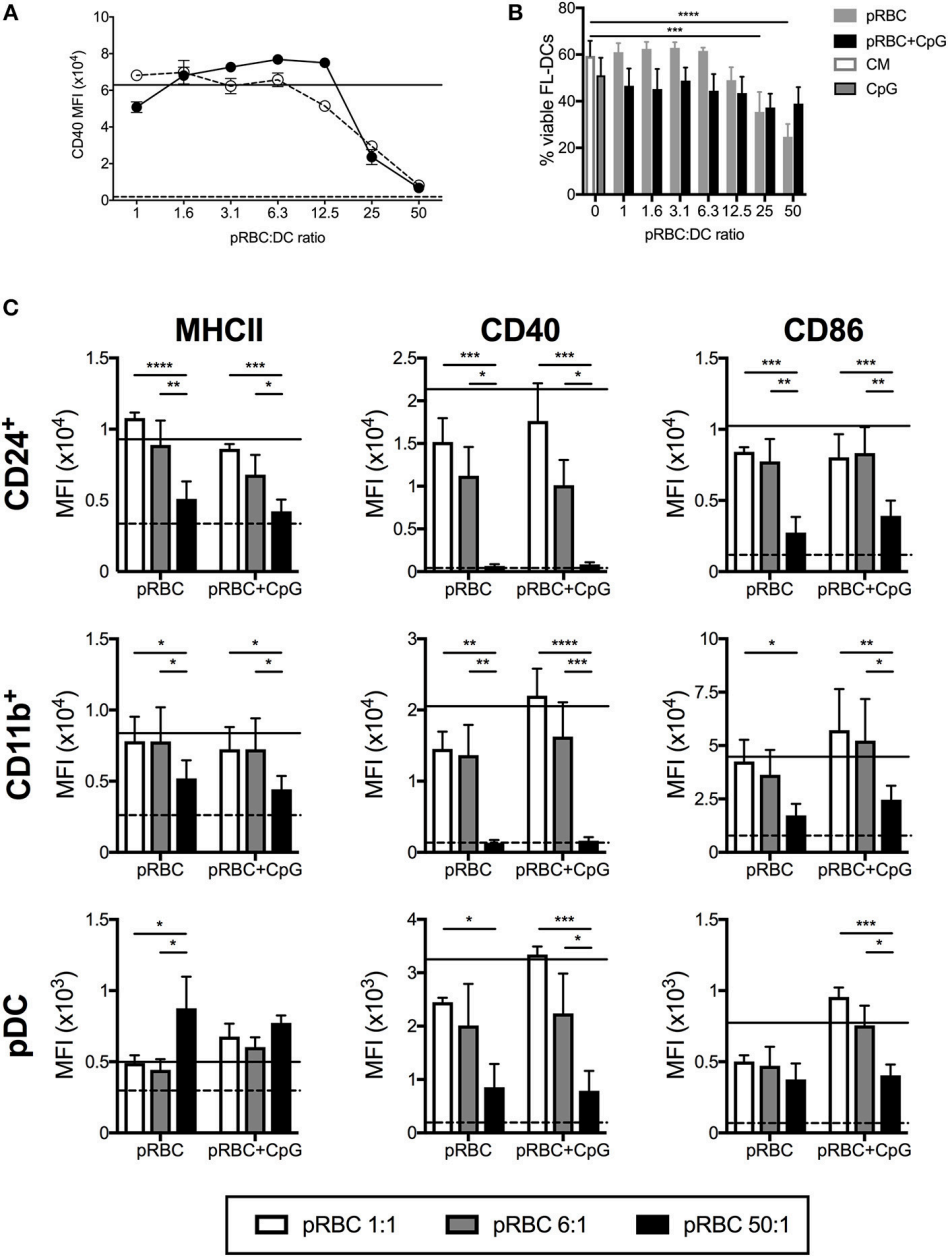
High doses of pRBCs induce poor FL-DC activation and prevent normal TLR9-mediated activation. **(A)** Expression of CD40 on CD24^+^ cDCs was downregulated in response to high doses of pRBCs alone (solid circles) or pRBCs + CpG2216 (hollow circles). Responses to CpG2216 alone (solid line) and uRBC alone (dashed line) are shown for comparison. **(B)** Viability of total FL-DCs in response to pRBCs alone (gray) or pRBCs + CpG2216 (black). Viability in response to culture medium alone (CM; white with gray border) or CpG2216 alone (CpG; gray with black border) are shown for comparison. **(C)** Expression of MHCII, CD40, and CD86 in response to pRBCs in CD24^+^ cDC, CD11b^+^ cDC, and pDCs. Co-stimulatory marker expression is shown in response to pRBC-to-DC ratios of 50:1 (black), 6:1 (gray), and 1:1 (white), as well as in response to CpG2216 (solid line) or uRBCs (dashed line). ^*^p < 0.05, ^**^p < 0.01, ^***^p < 0.001, ^****^p < 0.0001. *P*-values were determined by two-way ANOVA using multiple comparisons. Data shown is from three experiments, each experiment using pooled FL-DC cultures from two mice. Error bars represent SEM of three separate experiments. MFI—geometric mean fluorescence intensity.

To model the effect of different parasite concentrations on blood DC we analyzed the response of murine FL-DC to purified pRBCs from a high ratio of 50:1 to a low ratio of 1:1. In replicate, CpG2216 was added to the cultures to ask whether the presence of pRBCs affected the activation of FL-DC by an additional stimuli. The addition of uRBC alone were used to control for responses to RBC alone. To determine cell activation, MHCII and CD40 and CD86 co-stimulation molecule expression was measured upon FL-DCs (Figure 1). Analyses of total FL-DC indicated a reduction in CD40 expression at ratios of pRBC:DC of >12.5, and at lower concentrations in the additional presence of TLR9 ligand (CpG2216). Separate analyses of cDC and pDC subsets indicated that high pRBC concentrations led to a significant reduction of co-stimulation marker expression on both CD24^+^ and CD11b^+^ cDCs (Figure 1C) that was particularly strong for CD40, and also occurred in the additional presence of CpG2216 (Figure 1A). While DC viability decreased in response to 50:1 concentrations of pRBC alone, viability was not significantly decreased by pRBC in the presence of CpG yet co-stimulation molecule expression still decreased (Figure 1B). In response to low ratios of pRBCs of < 12.5 pRBC:DC, cDC expression of co-stimulatory markers was comparable to that induced by CpG2216.

A more complex relationship between MHCII and co-stimulatory marker expression and pRBC ratio was observed in pDCs. Upon stimulation with low ratios of pRBC, pDCs expressed MHCII, CD40, and CD86 at elevated levels compared to uRBCs alone, which could be further elevated in the presence of CpG2216. However, upon stimulation of pDC with pRBC ratios >25:1, in the presence or absence of CpG2216, CD40, and CD86 expression was reduced whereas MHCII (Figure 1C) and CD80 (data not shown) expression increased to levels above that seen with CpG2216 alone.

### The Ratio of pRBCs-to-DCs Alters FL-DC Patterns of Cytokine Production Including in the Presence of TLR9 Ligand

The data of Figure 1 indicated that at high pRBC:DC ratios, the response of DCs to CpG2216 was inhibited and that overall, DC activation was suppressed. To further investigate the activation of DC across a range of pRBC concentrations we analyzed the production of cytokines in the supernatants of the cultured FL-DC.

**Figure 2:**
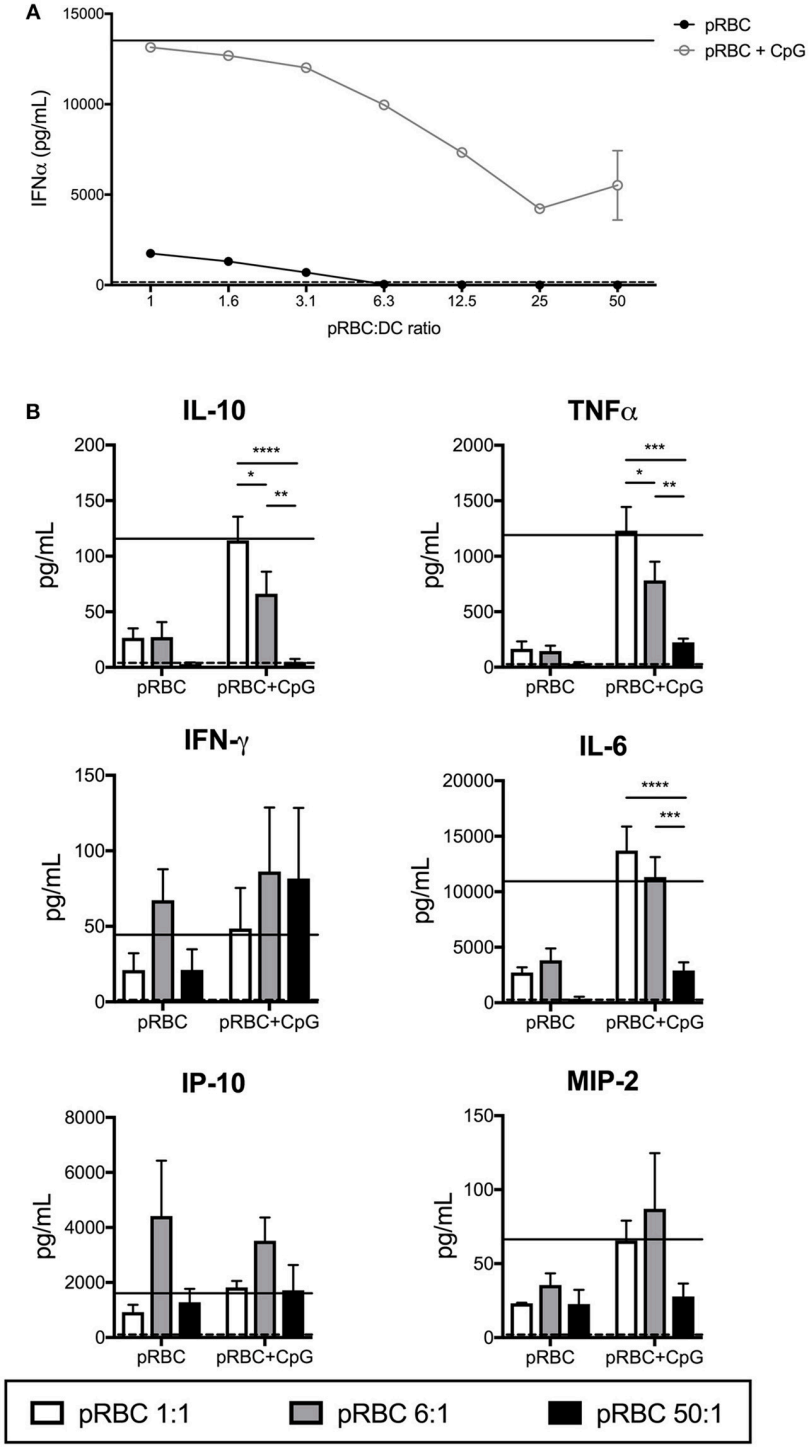
Production of cytokines by FL-DCs is inhibited by high pRBC-to-DC ratios. Supernatants from FL-DC cultures stimulated with pRBCs were assayed for cytokine production using FlowCytomix and ProcartaPlex multiplexes. **(A)** IFN-α expression by FL-DCs was suppressed by the presence of high ratios of pRBCs, even in the presence of TLR9 ligand (pRBC+CpG). **(B)** FL-DC production of cytokines in response to pRBC-to-DC ratios of 1:1 (white), 6:1 (gray), and 50:1 (black) alone, or in the presence of CpG2216. Cytokine expression in response to uRBCs (dashed line) or CpG2216 alone (solid line) are included for comparison. ^*^p < 0.05, ^**^p < 0.01, ^***^p < 0.001, ^****^p < 0.0001. *P*-values were determined by two-way ANOVA using multiple comparisons. Data shown is from three experiments with each experiment using pooled FL-DC cultures from two mice. Error bars represent SEM of three separate experiments.

Others have previously reported pDC-produced IFN-α in response to *P. falciparum* (19, 31, 32), with conflicting reports on whether the activation of pDCs was inhibited by pRBCs (19, 32). In the FL-DC cultures with pRBCs alone, IFN-α was only detectable in the cultures at ratios of < 12.5 parasites per DC (Figure 2A). At pRBC:DC ratios of 1:1 and 6:1, we detected high levels of IL-6 and IP-10 (CXCL10) and detectable levels of TNF-α, IL-10, IFN-γ, and MIP-2 (CXCL2, Figure 2B). At pRBC-to-DC ratios of 50:1, IL-6, TNF-α and IL-10 production were reduced to the baseline levels induced by uRBCs whilst IP-10, IFN-γ and MIP-2 were produced at levels similar to those observed with ratios of 1:1 pRBC:DC (Figure 2B).

We then investigated whether cytokine secretion to pRBCs was boosted in the presence of TLR9 ligand, CpG2216, or if pRBCs were able to suppress CpG2216-induced cytokine secretion. The pDC and pDC-like cells are the only cells capable of producing high levels of IFN-α to CpG oligonucleotides, potent TLR-9 ligands (33). Addition of CpG2216 to pRBC-DC co-incubations increased overall cytokine secretion but a pRBC dose-dependent suppression of TLR9-induced IFN-α, i.e., pDC responses, was observed (Figure 2A) from bulk cultures. Reduced production of both TNF-α and IL-10 were also observed with pRBC:DC ratios of 6:1 and above (Figure 2). This suppression was marked at pRBC:DC ratios of 50:1 for IL-10, TNF-α, IL-6, and MIP2 production (Figure 2B).

### Only Freshly Isolated pRBCs Are Suppressors of FL-DC Activation

**Figure 3:**
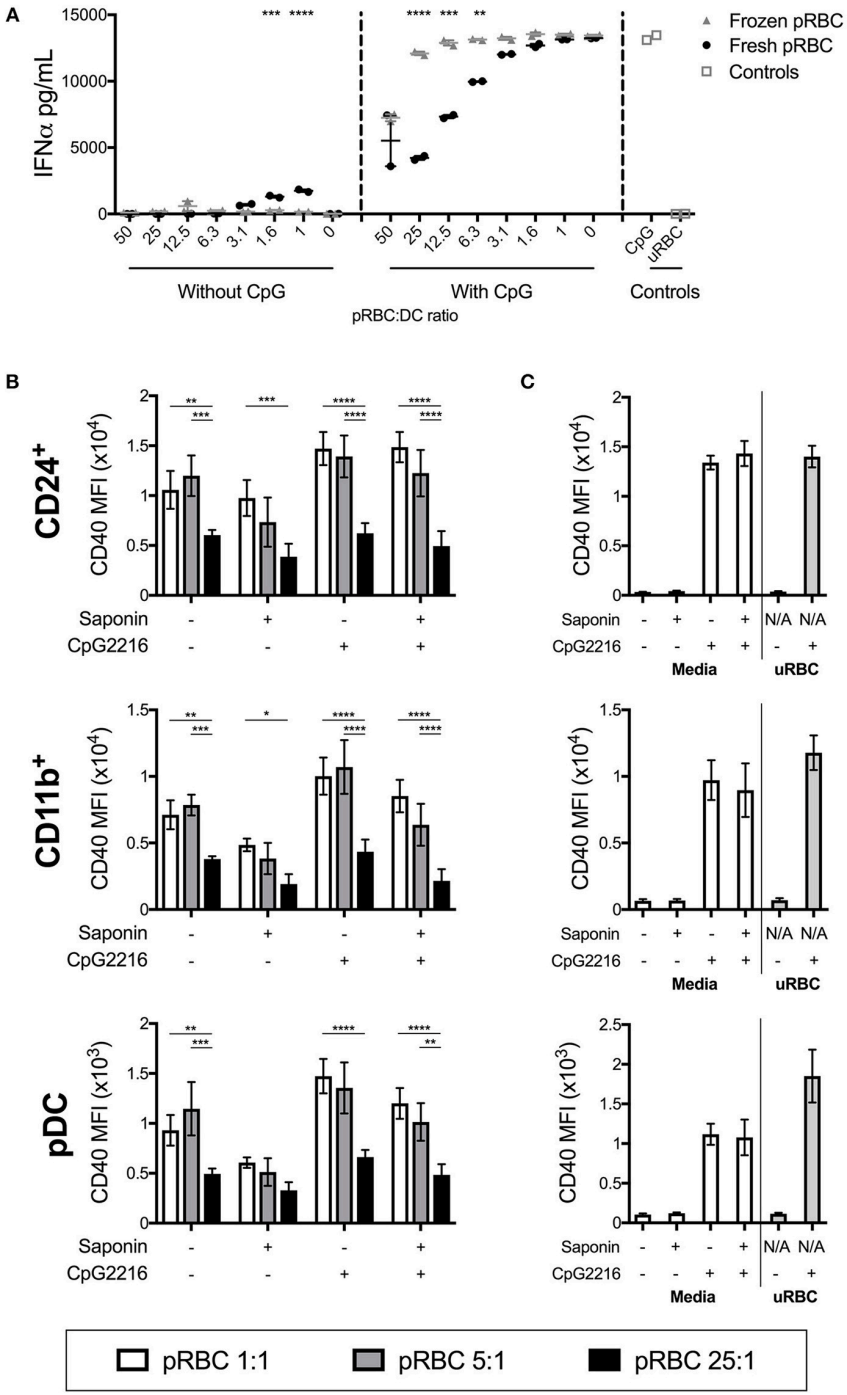
pRBC-induced suppression is dependent on fresh parasites but not on intact host RBC membranes. **(A)** A titration of frozen pRBCs were compared to freshly purified pRBCs for the ability to induce activation and parasite-induced suppression of FL-DC TLR9 responses. Shown are IFN-α responses either to parasites alone or in the additional presence of CpG2216. **(B)** FL-DCs were co-incubated with saponin-treated or untreated *P. falciparum* pRBCs for 18–20 h at three different parasite-to-DC ratios: 1:1 (white), 5:1 (gray), and 25:1 (black), in the presence or absence of CpG2216. DC subpopulations were gated and expression of CD40 is shown. Each bar shows the SEM of three separate experiments, with each experiment using FL-DC cultures derived from pooling two mice. ^*^p < 0.05, ^**^p < 0.01, ^***^p < 0.001, ^****^p < 0.0001. *P*-values were determined by two-way ANOVA using multiple comparisons. **(C)** To control for effects of any carry-over saponin, saponin was also added to empty tubes and washed alongside the pRBC samples. Culture medium alone or medium containing CpG2216 were aliquoted into these tubes (designated Saponin +) and used to stimulate FL-DCs. The lack of any suppression of CD40 expression by uRBCs alone (25:1) is shown.

We compared responses of FL-DCs stimulated with freshly isolated pRBCs and pRBCs previously purified and freeze-thawed. The protocol used for cryopreservation and thawing of pRBCs largely preserves pRBC integrity (34, 35) and is a convenient way of preparing standardized preparations of parasites for research, including experimental human infections (36, 37). However, frozen pRBCs were less efficient at activating the FL-DC (Figure 3A and data not shown). Moreover, they were inefficient at suppressing the TLR-9 activation of FL-DC compared to the freshly isolated pRBC (Figure 3A and Supplementary Figure 2). Therefore, frozen and fresh pRBC preparations were qualitatively different in their fundamental abilities to activate DCs.

### Removal of Host RBC Membrane Does Not Prevent Parasite-Induced Suppression of DC Responses

The differences in the ability of fresh vs. frozen and thawed pRBC preparations to affect FL-DC activation suggested that perhaps a reduction in pRBC membrane viability was the cause. To test this, we treated freshly isolated pRBCs with the detergent saponin. Saponin treatment of pRBCs disrupts the RBC membrane and the parasitophorous vacuolar membrane (38). Thus, saponin-treated trophozoites lack expression of malarial proteins normally located on the host red cell membrane, including molecules such as *Pf* EMP1 which have been postulated to inhibit innate immune responses (39–41).

Co-stimulatory marker expression by DCs was similar after stimulation with saponin-treated and untreated pRBCs, with pRBC:DC ratios of 1:1 and 5:1 actually inducing lower levels of activation whilst high doses still led to low CD40 upregulation. In the presence of CpG2216, DCs were similarly activated, regardless of saponin treatment, whilst high doses of saponin-treated pRBC still suppressed the CpG2216-driven CD40 (Figure 3) and CD86 upregulation (Supplementary Figures 3B,C) on cDC and pDC populations.

Thus, neither the presence of host RBC membrane nor surface-expressed parasite proteins, including *PfEMP1*, appeared to play a major role in suppression of FL-DC activation in the presence of high levels of parasites. Viability was comparable between FL-DCs exposed to saponin-treated and non-treated pRBCs for every ratio of pRBCs to DCs (Supplementary Figure 3A). This data also suggested that the differences in DC activation abilities between frozen and freshly isolated pRBC were intrinsic to the trophozoite and not dependent on the host RBC membranes.

### Merozoites Are Potent Stimulators of FL-DCs

**Figure 4:**
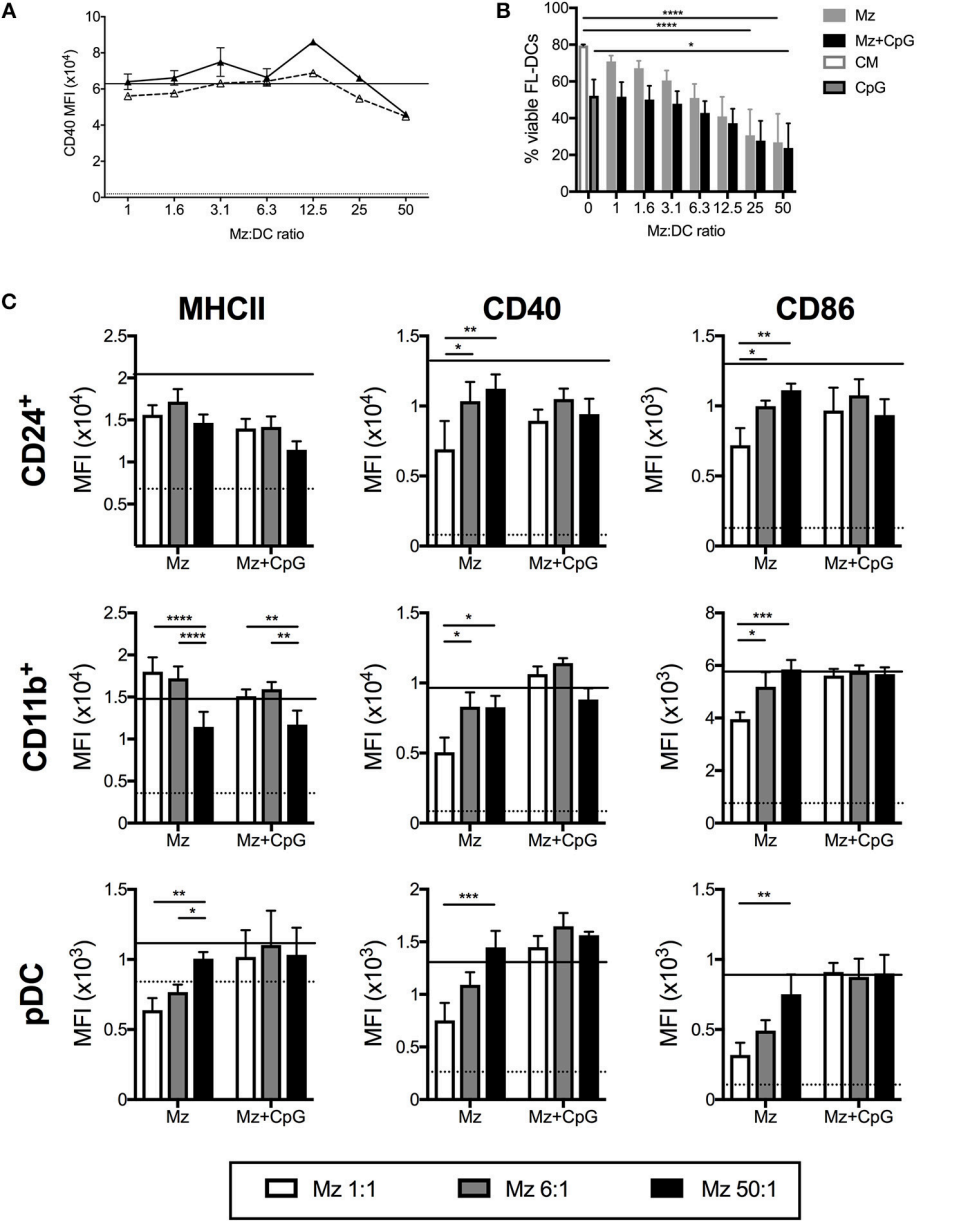
Merozoites induce potent activation but high cell death of FL-DC. **(A)** Surface expression of CD40 on CD24^+^ cDCs in response to 1–50 merozoites per DC (solid triangles) or in the additional presence of CpG2216 (hollow triangles). Level of CD40 in response to CpG2216 alone (solid line) and media alone (dotted line) are shown for comparison. **(B)** Exposure to high doses of merozoites results in death of up to 90% of FL-DCs after 18 h culture. Viability in response to culture medium alone (CM; white with gray border) or CpG2216 alone (CpG; gray with black border) are shown for comparison. **(C)** MHCII, CD40 and CD86 surface expression on FL-DCs gated for CD24^+^, CD11b^+^ or pDC in response to 1:1 (white bars), 6:1 (gray bars), or 50:1 (black bars) merozoites:DC, alone or in the addition presence of CpG2216.). ^*^p < 0.05, ^**^p < 0.01, ^***^p < 0.001, ^****^p < 0.0001. *P*-values were determined by two-way ANOVA using multiple comparisons. Data shown is from three experiments, each experiment using pooled FL-DC cultures from two mice. Error bars represent SEM of three separate experiments.

Merozoites are the extracellular life stage of the malaria parasite. FL-DCs stimulated with ratios of merozoites to DCs from a high ratio of 50:1 to a low ratio of 1:1 were, in the main, highly activated, with dose-dependent increases of all surface activation markers examined (Figure 4), although CD11b^+^ FL-DC showed reduced MHCII expression with high concentrations of merozoites (Figure 4C). However, in contrast to stimulation with pRBCs, at ratios higher than 12.5 merozoites:DC significant DC death was observed (Figure 4B). The rate of cell death at these ratios was high, consistent with excessive activation, and while cell surface activation of the remaining DC by TLR9 ligand was largely not perturbed by high concentrations of merozoites (Figure 4C), these results should be viewed in light of the very high rates of DC death at these parasite ratios.

### FL-DC Production of Cytokines in Response to Merozoites Generally Increases With Higher Merozoite Dose

**Figure 5:**
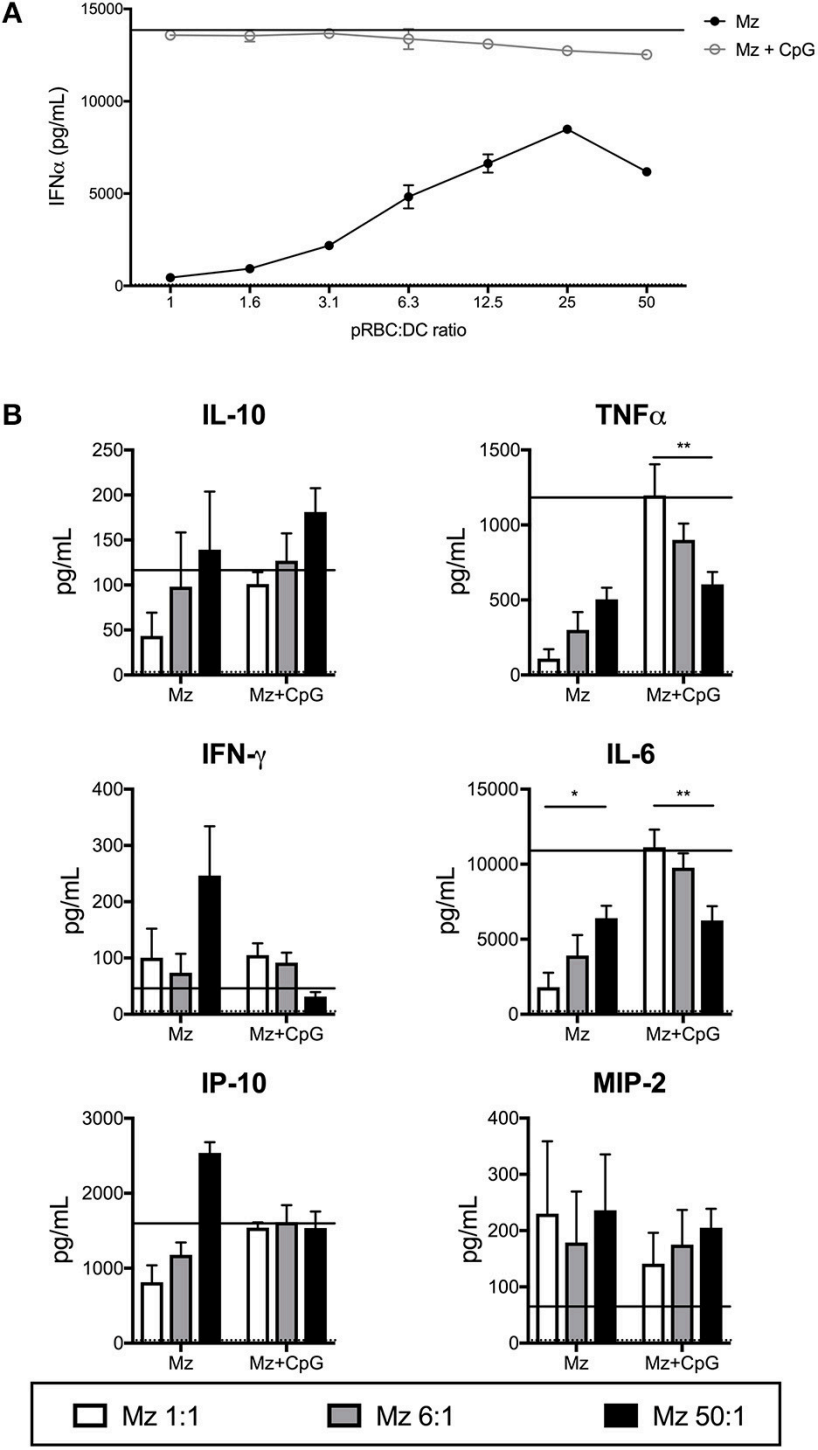
Merozoites induce expression of a range of cytokines from FL-DCs. FL-DCs were incubated for 18 h with merozoites (Mz) and cytokines were measured in the supernatant using FlowCytomix and ProcartaPlex multiplexes. **(A)** High ratios of Mz:DCs induce high expression of IFN-α. **(B)** Mz:DC ratios of 1:1 (white bars), 6:1 (gray bars), or 50:1 (black bars). The expression in response to media alone (dotted line) and CpG2216 alone (solid line) were included for comparison. ^*^p < 0.05, ^**^p < 0.01. *P*-values were determined by two-way ANOVA using multiple comparisons. Figure shows pooled data from three experiments of FL-DC cultures derived from two mice each.

In response to increasing doses of merozoites, FL-DCs produced increasing amounts of cytokines (Figures 5A,B). This was inverse to the effect observed with pRBCs (Figure 2). Merozoites were a potent stimulus for IFN-α production. In the presence of CpG2216, production of TNF-α and IL-6 was reduced with increasing merozoites, potentially due to high merozoites concentrations inducing high DC death (Figure 4B). Overall, merozoites appeared to be potent activators of FL-DCs, inducing high levels of cell death at the highest concentrations but apparently not suppressing FL-DC activation by TLR9 ligands.

### Merozoites Cannot Rescue FL-DC Suppression Induced by pRBCs

**Figure 6:**
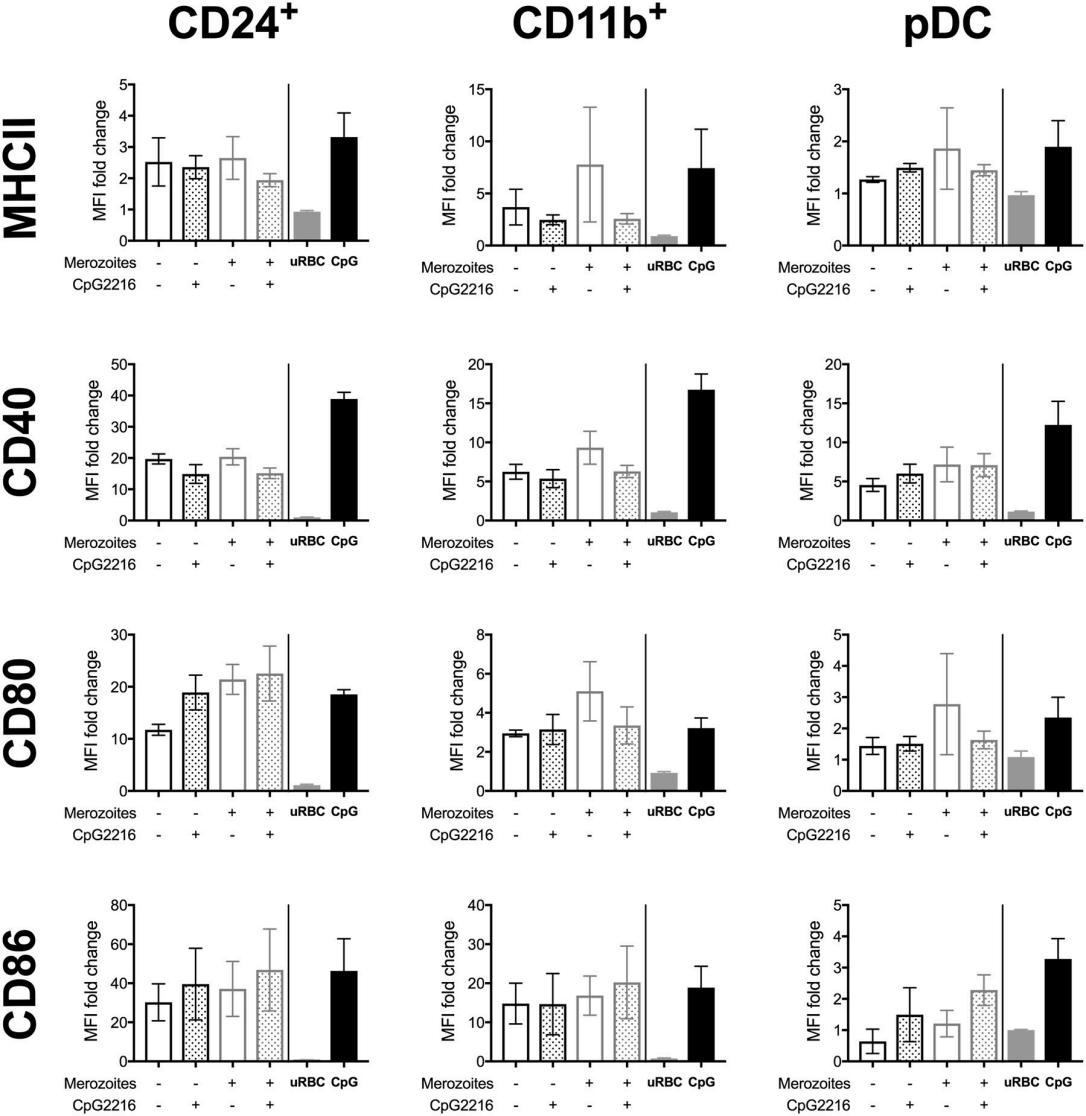
Stimulation with mixed high-dose pRBCs and merozoites in the presence or absence of CpG2216 does not result in recovery of CD40 expression. FL-DCs were stimulated with either pRBCs alone, or pRBCs in the presence of an equivalent ratio of merozoites (Mz), in the presence or absence of CpG2216. All pRBCs and merozoites were added at a ratio of 25 per DC. Neither the addition of merozoites nor the addition of CpG2216 resulted in rescue of CD40 expression, but other markers demonstrated a heterogenous response. Co-stimulatory marker expression is represented as fold increase over media control and each bar represents mean and SEM of two experiments based on pooled FL-DC cultures of two mice.

During malaria infection DCs would encounter both trophozoite and merozoite life-stages of the malaria parasite. Since merozoites did not suppress DC activation we tested whether merozoites would rescue the upregulation of CD40 in the presence of pRBCs, with or without TLR9 ligand. A ratio of 25 pRBCs:DCs was used with the addition of equal numbers of merozoites. Ratios of 25 parasites per DC for each life stage were chosen as concentrations that were still inhibitory for pRBCs (Figure 1A) but maintained high CD40 levels in response to merozoites alone (Figure 4A). Whilst merozoites are potent stimulators of MHC and co-stimulatory molecule expression on FL-DCs (Figure 4), they were unable to efficiently activate FL-DCs in the presence of pRBCs, particularly the upregulation of CD40 (Figure 6), either when mixed with pRBCs alone or in the additional presence of CpG2216 (Figure 6).

## Discussion

We have shown that at doses of >6 parasites to 1 DC, *P. falciparum* pigmented trophozoites possess a stage-specific capacity to suppress specific DC functions. This suppressive capacity particularly affects the upregulation of CD40 on all DC subsets and the ability of DCs to produce key cytokines; IFN-α, TNF-α, IL-6, and IL-10. This suppressive capacity of pRBCs is not dependent upon *Pf* EMP1 or other parasite molecules expressed on, or other properties of, the infected host RBC membrane (Figure 3).

The pRBCs not only suppressed DC responses to themselves at ratios of >6 per DC but also suppressed the ability of DCs to respond to the potent TLR9 ligand, CpG2216 (Figure 1). This included inhibiting the ability of pDCs to produce IFN-α to CpG2216 (Figure 2A). Although suppressing TLR9-mediated DC activation, increasing doses of pRBCs did not enhance DC death. Others have previously shown that monocyte-derived DCs, DCs fundamentally different to those investigated herein, also do not require *Pf* EMP1 to be suppressed, though suppression of DC activation at doses of 100:1 were at least in part due to DC apoptosis (42). The data shown herein of suppression of TLR9-mediated FL-DC activation by pRBCs is independent of cell death (Figure 1). In the aforementioned study, it was also shown via transwell experiments that cell-cell contact was not required for 100:1 doses of pRBCs to inhibit TLR-mediated upregulation of HLA-DR, CD83, and CD86 (42). Similarly, lysates from pRBCs induced chemokine production from human DCs, similarly to intact pRBCs (32). Both suppression and activation of DCs by pRBCs are therefore mediated by soluble factors, which could potentially be addressed by co-incubating TLR-stimulated DCs with conditioned parasite medium and measuring suppression.

In stark contrast, purified merozoites induced dose-responsive activation of the FL-DCs and high (likely activation-induced) DC death at high doses (Figures 4, 5). The merozoites were not able to rescue the activation of FL-DCs in the presence of pRBCs; in fact, pRBCs inhibited the strong merozoite-driven DC activation (Figure 6). DC death in malaria is via the apoptotic pathway (4, 42, 43), and it is unclear whether the DC death observed in response to high ratios of parasites in this study is due to parasites directly activating pro-apoptotic signals or whether it is apoptosis as a natural endpoint of DC activation. The increased DC apoptosis may inhibit activation of surrounding DCs by impairing the maturation of immature DCs (44) and polarizing DCs toward a tolerising phenotype (45). Regardless, DC apoptosis is likely a major contributor to the short duration of immune memory to malaria. Determining whether apoptosis is driven by maturation or parasites will help to identify methods of reducing apoptosis and improve downstream DC responses.

In contrast, ratios of 1:1 pRBC potently activated DCs to upregulate co-stimulatory markers (Figure 1) and cytokines, including IFN-α (Figure 3). Merozoites on the other hand, really showed a dose response effect in their ability to activate DC (Figures 4, 5), although ratios of 1 merozoite per DC still activated DC, highest levels of cytokines and cell surface markers were reached with more than 6 merozoites per DC. As merozoites and trophozoites have divergent transcriptomes (46, 47), this may help to identify potential activatory proteins. Furthermore, DC activation by these very low ratios of parasites suggests that even very low parasitaemia may induce potent DC activation, at least initially. Asymptomatic parasitaemia, often seen in areas of high transmission, may reflect a pattern where initial potent DC-driven responses are suppressed by subsequent high-parasitaemia infections, leading to immunosuppression and tolerance. In contrast, low-parasitaemia infections may actually induce potent DC responses that lead to the generation of memory.

As regards physiological relevance of the parasite-to-DC ratios examined in this study, there is relatively little information on bioavailability of blood-stage malaria parasites to splenocytes *in vivo*. However, *in vitro* studies suggest that in high-parasitaemia settings a large proportion of merozoites fail to invade (26). This would result in high volumes of merozoites circulating to or being trafficked to the primary lymphoid tissues by migratory DCs, potentially even to the 50:1 ratios reported in this study. It is also observed *in vivo* that the spleen can remove parasites from RBCs without inducing RBC lysis (48–50), and while there is no information at what rate this occurs, splenocytes may encounter membrane-free pRBC at a rate dependent on overall parasite burden. Potentially, splenic DCs may encounter very high ratios of membrane-free pRBC in high parasitaemia settings, and as DC responses are not significantly affected by pRBC membrane, it is possible that the patterns reported in our study reflect physiological phenomena occurring in lymphoid organs.

An important point revealed in this work was that the DC response to frozen and thawed pRBCs is distinct from the response to fresh pRBCs (Figure 3). Methods to standardize frozen pRBC preparations have been used to prepare parasite vaccinations for standardization of controlled human malaria infection trials in naïve individuals (36). While this method induces viable parasites, it is likely that a proportion of ring-stage trophozoites would not survive the freezing process and may lead to induction of sub-optimal DC responses or tolerance. Furthermore, it serves as a caution for *in vitro* experiments that may use cryopreserved pRBCs as an immune stimulus. The data shown would argue that a comparison with freshly isolated pRBCs is needed to determine whether responses of human DCs and other innate responses *in vivo* and *in vitro* are qualitatively different when comparing inoculation with freeze-thawed vs. fresh pRBC preparations.

The surface activation marker most consistently suppressed by the presence of pRBCs, in both cDCs and pDCs, was CD40. Ligation of CD40 is crucial as it improves DC survival and enhances antigen presentation, therefore acting as a direct marker for DC activation (3). The interaction of CD40:CD40L between DCs and T cells activates both cell types and provides a vital positive feedback loop for cytokine secretion and co-stimulatory marker upregulation by DCs (3). Loss of CD40 on the surface of pRBC-exposed DCs could result in failure to induce downstream co-stimulatory pathways, resulting in overall failure of DC function. Work by Mukherjee and Chauhan highlights the importance of CD40 signaling for upregulation of other co-stimulatory markers in the moDC:*P. falciparum* context. When CD40L was added to moDCs co-incubated with “suppressive” doses of pRBCs, expression of CD83 was unaltered and expression of CD80 enhanced, rather than suppressed (51). A similar mechanism may be present in *bona fide* DCs. If CD40 expression is restored in DCs exposed to high doses of *P. falciparum*, it may restore their ability to stimulate T and B lymphocytes, which has implications for the design of next generation vaccine adjuvants that either target pRBC mechanisms of CD40 down-regulation or independently lead to enhanced expression of CD40 on DCs.

Murine FL-DC were used as a model for human blood DCs as they could be easily generated in large numbers and consistently produce similar levels of pDCs and cDCs (Supplementary Figure 4). FL-DCs also show greater transcriptomic similarities to their human counterparts than moDCs, another model commonly used to study malaria-DC interactions (9). The major difference between murine FL-DCs and human blood DCs is that only pDCs in humans express TLR9 (52). However, findings in human moDCs, which also do not express TLR9, are also suppressed by high doses of pRBCS (42, 53), suggesting high pRBC doses can suppress DC activation by multiple alternate pathways.

The mechanism by which pRBCs inhibit DC activation remains unclear. The effects of hemozoin upon DCs have been debated in the literature (18, 19) but it is unlikely to be the suppressive factor in this model as merozoite preparations were not hemozoin-depleted prior to DC co-incubation, yet still induced potent DC activation. A possible explanation may be found in the proteomic and transcriptomic differences between pRBCs and merozoites. The merozoite transcriptome is 45% smaller than that of the trophozoite. Merozoites lack the expression of many proteins required for hemoglobin digestion, protein export, and parasite metabolism (46, 47). One of these trophozoite-restricted protein families may contain the factor responsible for suppression; alternately, it may be a lipid, RNA byproduct or metabolite of the pigmented trophozoite, which is a very metabolically active life stage.

In conclusion, we demonstrate that pRBCs can suppress specific key DC functions and activation, in contrast to the activatory effect of merozoites. While DCs would interact with both merozoites and pRBCs in the blood, the specific suppressive effect of pRBCs appeared dominant and occurred even in the presence of merozoites. These findings may help partly explain why malaria immunity is typically slow to be acquired through natural exposure, why vaccine efficacy and vaccine-induced immune responses are lower in malaria endemic settings than among malaria-naïve subjects (54), and why immunity to malaria is generally not well-maintained (55). Further research to understand the mechanistic basis for these effects and identifying strategies to overcome them may be crucial for the development of highly efficacious and long-lasting vaccines.

## Ethics Statement

This study was carried out in accordance with NHMRC guidelines and approved by the Animal Ethics Committee at AMREP Animal Services Precinct Animal Centre, Melbourne, Australia and Monash University, Clayton, Australia.

## Author Contributions

XZY and RJL carried out the research and analyzed the data. GF carried out essential experiments. JP contributed essential technical expertise. MOK and JGB designed and supervised the research and analyzed the data. XZY wrote the first draft. All authors contributed to writing of the manuscript.

### Conflict of Interest Statement

The authors declare that the research was conducted in the absence of any commercial or financial relationships that could be construed as a potential conflict of interest.
