# Supplementary material for: Different Life Cycle Stages of Plasmodium falciparum Induce Contrasting Responses in Dendritic Cells
Source: Front Immunol. 2019 Jan 31;10:32. doi: 10.3389/fimmu.2019.00032 (PMC6365426; doi:10.3389/fimmu.2019.00032)
Supplement: Supplementary file 1 [file Data_Sheet_1.pdf]

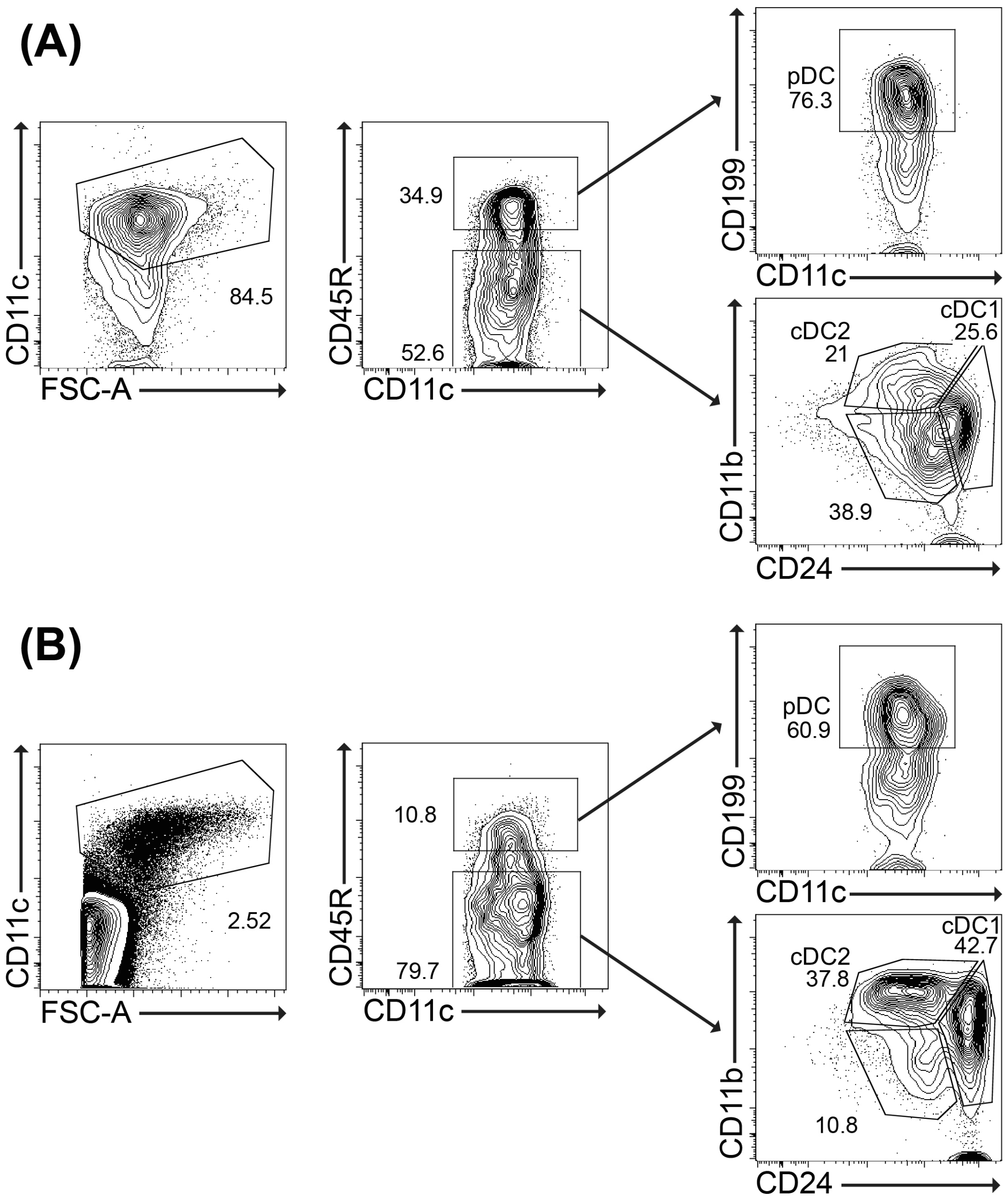

SUPPLEMENTARY FIGURE 1: Flow cytometric gating strategy for identification of FL-DC subsets.

Gating strategy is shown for (A) FL-DCs alone or (B) FL-DCs in the presence of pRBCs, where there was a much lower percentage of FL-DCs due to the large amount of pRBC in the preparation. Live, single cells of medium size and granularity were gated and three FL-DC subsets were identified based on expression of CD11c, CD11b, CD24, CD199, and CD45R: pDCs ($CD11c^{+}CD45R^{+}CD199^{+}CD11b^{-}CD24^{-}$),

CD24^{+} cDCs (CD11c^{+}CD45R^{-} CD11b^{lo}CD24^{+}), and CD11b^{+} cDCs (CD11c^{+}CD45R^{-} CD11b^{hi}CD24^{lo}). Another preDC population (CD11c^{+}CD45R^{mid} CD11b^{lo}CD24^{lo}) was also identified but was not a focus of this report. Numbers denote the percentage of cells within each gate.

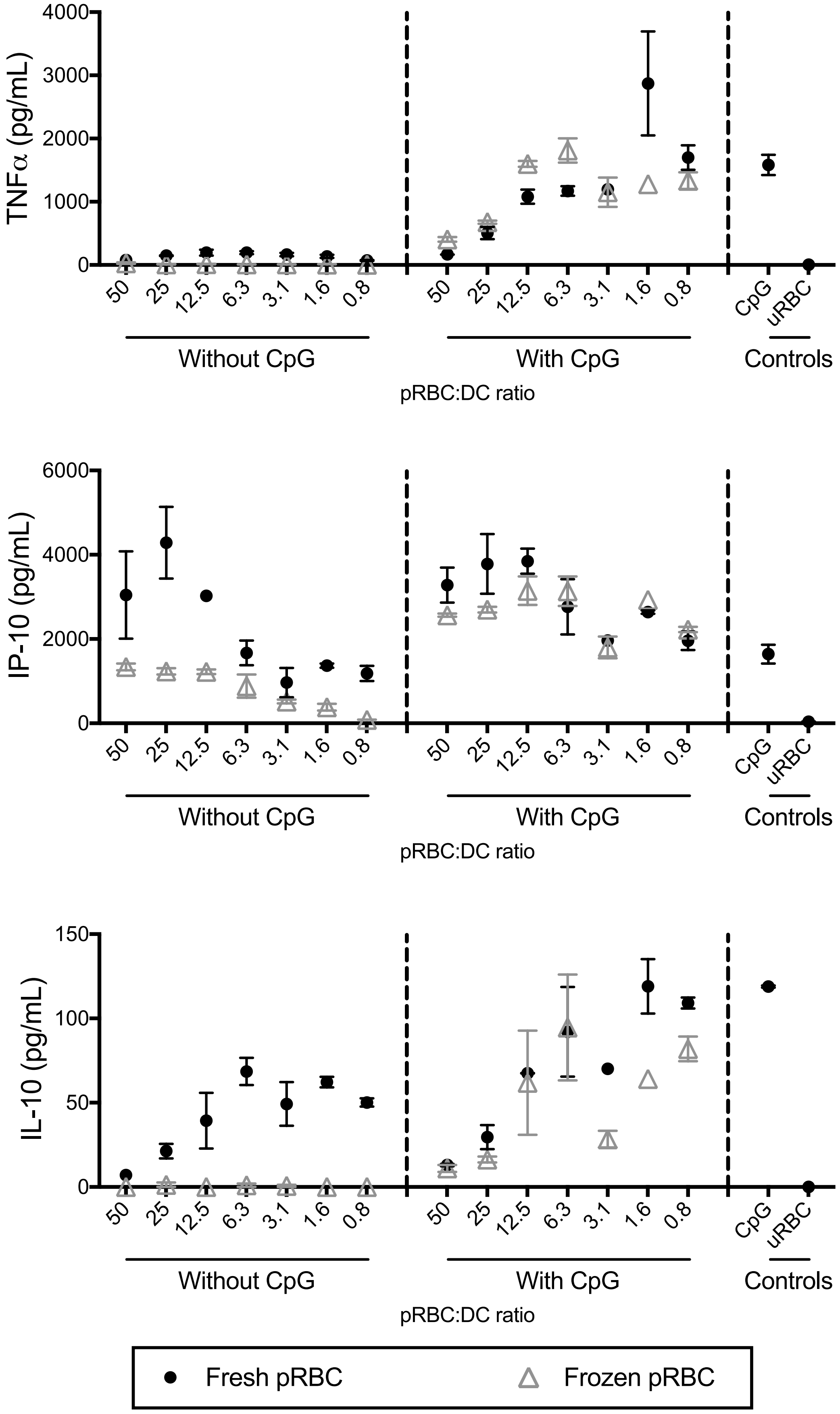

TNFα (pg/mL)
0
1000
2000
3000
4000
IP-10 (pg/mL)
0
2000
4000
6000
IL-10 (pg/mL)
0
50
100
150
50
25
12.5
6.3
3.1
1.6
0.8
CpG
uRBC
Without CpG
With CpG
Controls
pRBC:DC ratio
Fresh pRBC
Frozen pRBC

SUPPLEMENTARY FIGURE 2: Differences in cytokine production induced by fresh versus freeze-thawed pRBCs.

FL-DCs stimulated with pigmented trophozoites purified on the same day ('Fresh', black circles) as opposed to trophozoites that had been previously purified, then frozen and thawed ('Frozen', grey triangles), exhibited different patterns of cytokine production, with frozen pRBCs consistently inducing lower or undetectable levels of cytokines. However, upon addition of CpG2216 similar patterns were observed between frozen and fresh pRBCs. Each data point represents pooled replicates from a single experiment.

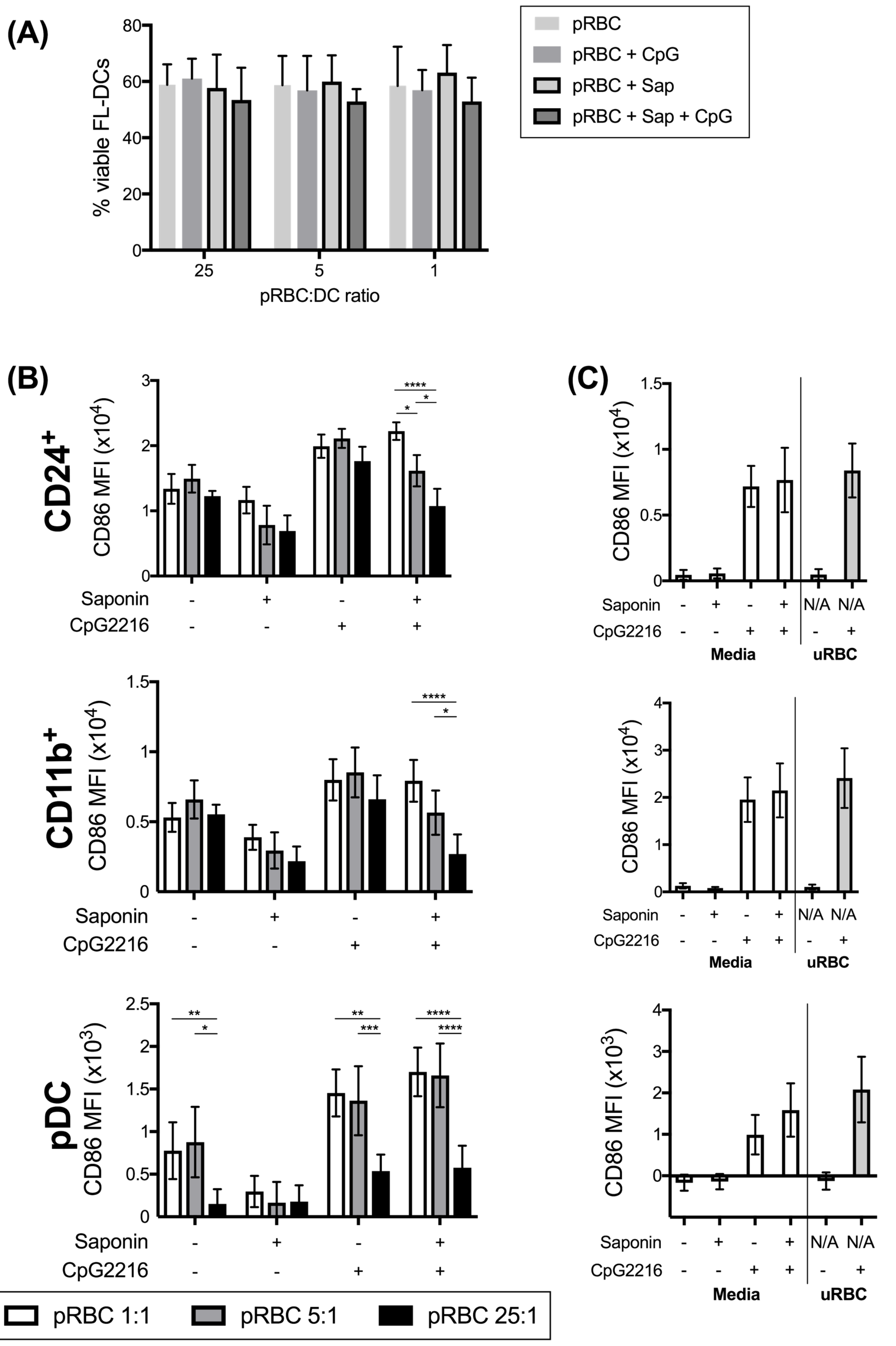

SUPPLEMENTARY FIGURE 3: Saponin treatment does not abolish the ability of pRBC to suppress DC function.

Parasitised RBCs (pRBCs) treated with saponin to remove host and parasitophorous vacuole membranes were added to FL-DCs. FL-DC activation was measured after 18-20h. FL-DC viability (A) and upregulation of CD86 (B) was not negatively affected by saponin treatment and was comparable to FL-DCs exposed to untreated pRBCs in the presence or absence of CpG2216. Data from non-pRBC controls (C) is also shown for comparison. Data shown is mean and range of three individual experiments with each experiment using FL-DC cultures pooled from two mice.

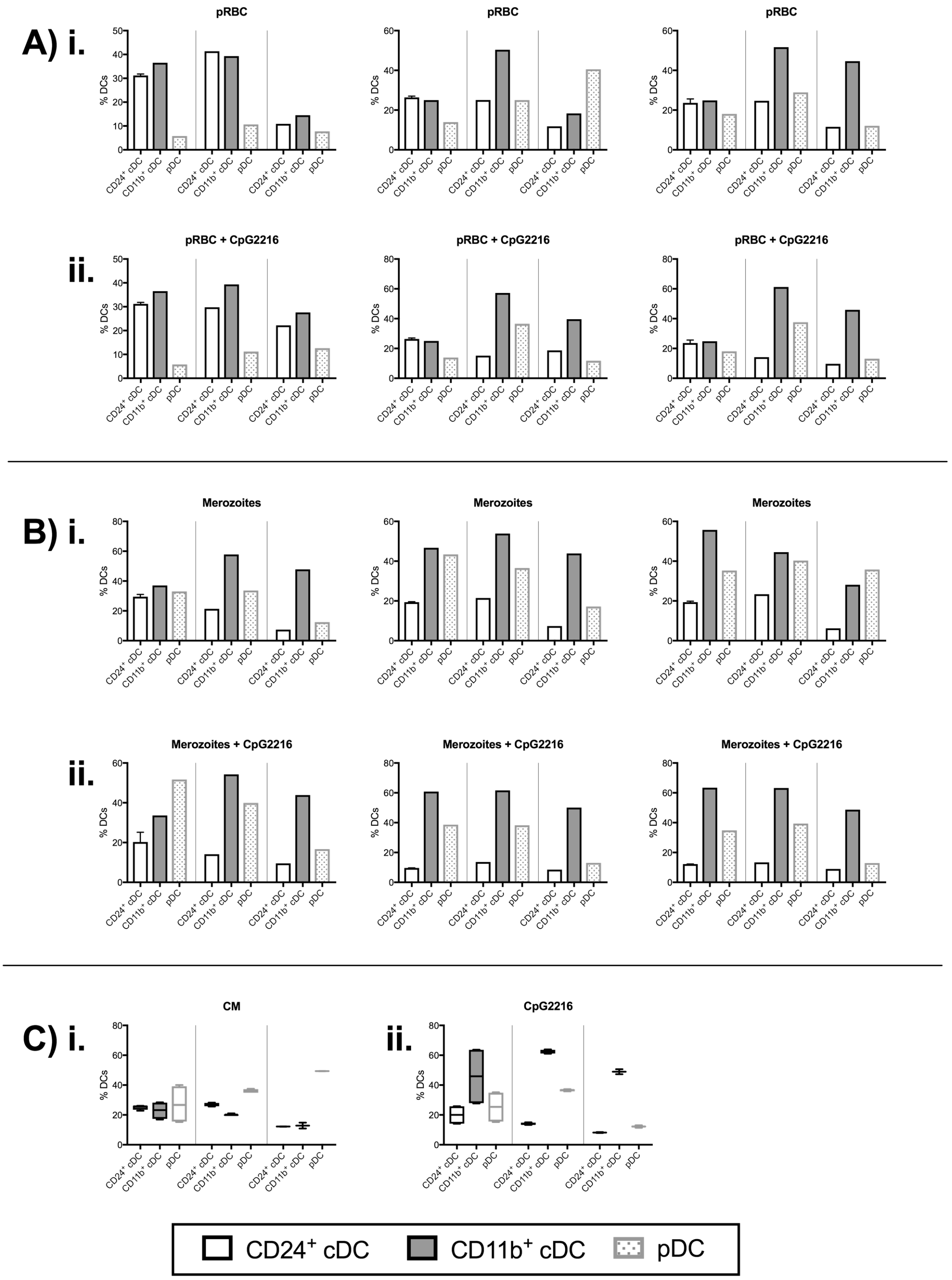

SUPPLEMENTARY FIGURE 4: Proportions of DC subsets per experiment. DC proportions are shown as a percentage of parent, with each box and whisker plot showing range and median of replicates for each experiment. Proportions of $CD24^+$ (black border, clear fill) and $CD11b^+$ (black border, grey fill) cDCs and pDCs (grey border, clear fill) are shown for (A) pRBC and (B) merozoite stimulations in the (i) absence or (ii) presence of TLR9 ligand. (C) Proportions of DC in response to (i) culture media and (ii) TLR9 ligand are shown for comparison.

Marker	Clone	Fluorochrome	Source
CD11b	M1/70	FITC	BD
		PE-CF594	BD
CD11c	HL3	PerCPCy5.5	BD
CD24	M1/69	eF450	eBioscience
CD40	3/23	FITC	BD
		APC	BD
CD45R (B220)	RA3-6B2	APC-Cy7	BD
CD69	H1.2F3	eF605	eBioscience
		PE	BD
CD80	16-10A1	BV650	BioLegend
CD86	GL1	A700	BD
CD172α (Sirp-α)	P84	APC	eBioscience
CD199 (CCR9)	CW-1.2	PE-Cy7	Biolegend
	eBioCW-1.2	PE	eBioscience
CD205 (DEC205)	205yekta	PE-Cy7	eBioscience
MHCII	M5/114.15.2	V500	BD
Live/dead		PI	Sigma Aldrich
		Live/Dead Aqua	Invitrogen

SUPPLEMENTARY TABLE 1: Fluorescence-conjugated antibodies and viability stains for use in flow cytometry.

Panel design was carried out by use of the BioLegend Spectral Analyser. Single-colour compensations were carried out using cells or BD CompBeads (BD) at times when cell numbers were insufficient. All cell staining was carried out at the above pre-titrated optimal dilutions in 1X MT PBS containing 2% v/v FCS and 10% v/v 0.1M EDTA and analysed using a BD LSRII or BD Fortessa.
